# *Bacillus velezensis* YXDHD1-7 Prevents Early Blight Disease by Promoting Growth and Enhancing Defense Enzyme Activities in Tomato Plants

**DOI:** 10.3390/microorganisms12050921

**Published:** 2024-04-30

**Authors:** Wangxi Li, Lili Sun, Hangtao Wu, Wenjie Gu, Yusheng Lu, Chong Liu, Jiexin Zhang, Wanling Li, Changmin Zhou, Haoyang Geng, Yaying Li, Huanlong Peng, Chaohong Shi, Dan Wang, Guixiang Peng

**Affiliations:** 1 College of Natural Resources and Environment, South China Agricultural University, Guangzhou 510642, China; liwangxi2021@stu.scau.edu.cn; 2 Institute of Agricultural Resources and Environment, Guangdong Academy of Agricultural Sciences, Key Laboratory of Plant Nutrition and Fertilizer in South Region, Ministry of Agriculture, Key Laboratory of Nutrient Cycling and Farmland Conservation of Guangdong Province, Guangzhou 510640, China; sunlili@gdaas.cn (L.S.); wuhangtao@gdaas.cn (H.W.); guwenjie@gdaas.cn (W.G.); luyusheng1@gdaas.cn (Y.L.); liuchong1990@gdaas.cn (C.L.); zjiexin@outlook.com (J.Z.); liwanling@gdaas.cn (W.L.); zhouchangmin@gdaas.cn (C.Z.); ghy741858672@163.com (H.G.); liyaying@gdaas.cn (Y.L.); penghuanlong@gdaas.cn (H.P.); shichaohong@gdaas.cn (C.S.)

**Keywords:** biocontrol, *Bacillus velezensis*, tomato early blight, genome

## Abstract

*Bacillus velezensis* is well known as a plant growth-promoting rhizobacteria (PGPR) and biocontrol agent. Nevertheless, there are very few reports on the study of *B. velezensis* on tomato early blight, especially the biocontrol effects among different inoculation concentrations. In this study, an IAA-producing strain, *Bacillus velezensis* YXDHD1-7 was isolated from the tomato rhizosphere soil, which had the strongest inhibitory effect against *Alternaria solani*. Inoculation with bacterial suspensions of this strain promoted the growth of tomato seedlings effectively. Furthermore, inoculations at 10^6^, 10^7^, and 10^8^ cfu/mL resulted in control efficacies of 100%, 83.15%, and 69.90%, respectively. Genome sequencing showed that it possesses 22 gene clusters associated with the synthesis of antimicrobial metabolites and genes that are involved in the production of IAA. Furthermore, it may be able to produce spermidine and volatile compounds that also enhance plant growth and defense responses. Our results suggest that strain YXDHD1-7 prevents early blight disease by promoting growth and enhancing the defense enzyme activities in tomato plants. This strain is a promising candidate for an excellent microbial inoculant that can be used to enhance tomato production.

## 1. Introduction

Tomatoes (*Solanum lycopersicum* L.) are widely appreciated by consumers because of their high nutritional value and good taste. Early blight causes about 80–86% in tomato yield losses and consequently receives considerable attention worldwide [1]. The pathogen *Alternaria solani* has a fast infection rate and mainly targets tomato plants at the seedling and vegetative growth stages [2]. High temperatures and humidity will accelerate early blight outbreaks [3]. At present, this disease is mainly prevented and controlled through the use of chemical pesticides, grafting, and the breeding of disease-resistant cultivars. However, due to the high cost of the latter method and the difficulty in promoting it, it is difficult to breed disease-resistant cultivars for production. The long-term use of chemical pesticides causes increased resistance to pathogenic bacteria and serious environmental pollution, leading to a series of environmental issues [4]. Therefore, it is urgent to develop an efficient, green, safe, and non-toxic control measure for the prevention and control of tomato early blight. As chemical fungicides and resistant cultivars, which have an unstable polygenic inheritance, are not environmentally friendly, biocontrol is considered a suitable and sustainable alternative [5,6].

Biocontrol technology based on microorganisms has been widely used in developed countries because it has a low cost, is environmentally friendly, and does not result in the production of chemical residues [7]. In recent years, microorganisms have been recognized as alternative tools for the prevention of plant diseases and growth promotion [8]. Until now, the most widely reported biocontrol agents (BCA) against *A. solani* mainly belonged to genera *Bacillus*, *Pseudomonas*, and *Trichoderma* [9,10]. These BCAs have been reported to inhibit pathogen growth or triggering resistance in tomato plants [11,12]. Among them, *Bacillus* spp. have been widely used for the biological control of phytopathogens, as they have the advantages of rapid reproduction, strong adaptability, and broad-spectrum bacteriostatic properties [13]. The *B. amyloliquefaciens* strain XJ5 has been shown to significantly inhibit the conidial germination and alter the mycelial morphology of *A. solani* [14]. Moreover, fengycins released from the *B. subtilis* strain ZD01 were shown as the main antifungal lipopeptide substances capable of strongly reducing the pathogenicity of *A. solani* by inhibiting conidial germination. Another study showed a significant control effect of the *B. velezensis* strain HY19 on the incidence of gray mold in tomato, which was reduced by 73.12–76.51% [15]. *Bacillus* spp. represent a potential reservoir of high-quality biocontrol compounds, and further research is expected to discover more strains with these properties. However, only a few studies have been conducted on the effects of *B. velezensis* on tomato early blight. Furthermore, genome sequencing and analysis associated with antimicrobial compounds or PGPR properties are extremely helpful for deep insights into functional strains [16].

Recent study proved that microbes that are beneficial for plants such as *Bacillus* that raise the reactive oxygen species (ROS) level of the host to prevent pathogen attacks, and this may contribute to forming disease-suppressive soil [17]. *Bacillus* was reported to release volatile compounds to significantly increase the activities of host defense enzymes such as peroxidase (POD), polyphenol oxidase (PPO) and phenylalanine deaminase (PAL) [18,19,20]. POD catalyzes the hydrolysis of H_2_O_2_ to produce H_2_O and O_2_ [21], and PPO catalyzes the formation of lignin and phenols in plant cells, thereby reducing the accumulation of ROS [22]. PAL is involved in the biosynthesis of salicylic acid (SA) and plays an important role in plant defense [23]. Thus, obtaining biocontrol resources with the ability to enhance plant antioxidant and defense enzyme activities is important for the prevention and control of soil-borne diseases. In the present study, we isolated a strain with strong antagonistic activity against *A. solani* from the rhizosphere soil of healthy tomato plants grown for over 10 years in Guangdong Province and confirmed that it can not only promote plant growth but also inhibit tomato early blight in pot-grown plants. Furthermore, we identified its gene clusters associated with plant growth-promoting effects and antibiotic synthesis to further elucidate its characteristics.

## 2. Materials and Methods

### 2.1. Strain Isolation

The tomato rhizosphere soil was obtained from root surfaces (0.5–5 mm) in a field situated in Yangjiang City, Guangdong province, and they were taken to the laboratory in sterile bags for further analysis. A total of 10 g of soil sample was mixed with 90 mL of sterile water and incubated in a shaker at 180 rpm for 30 min. Subsequently, the supernatant was obtained and serially diluted from 10^−4^ to 10^−6^. A total of 200 μL of each dilution was spread on nutrient agar or Gauze’s synthetic agar medium No.1, and each dilution gradient was performed in triplicate. The samples were incubated at 30 °C for 2 days, and a single colony was selected for purification.

### 2.2. Screening and Identification of Antagonistic Strains

*Alternaria solani* ACCC 37,458 was purchased from the Agricultural Culture Collection of China, while *Ralstonia solanacearum* (from the host plant tomato or pepper), *Fusarium oxysporum* f.sp. *momordicae*, and *F. oxysporum* f.sp. *cubense* were isolated from Guangdong province. The fungal pathogens were cultured on potato dextrose agar media. After being cultured for 7 days at 28 °C, a 6 mm diameter pathogenic agar block was placed in the center of a new PDA agar plate. Meanwhile, the antagonistic strain was cultured on nutrient agar media for 24 h, then 6 mm agar blocks were cut out and placed 2.5 cm from the center. The radial mycelial growth of the fungal pathogen was measured after 7 days of incubation. The following formula was used for the calculation of the inhibition rate (%) [24]:

$$\mathrm{Inhibiton}\;\mathrm{rate}\;\left(\%\right)=\frac{R-r}{R}$$

where *R* is the mycelia radial growth on the control fungal plate and r is mycelia radial growth on the antagonistic strain-treated plate.

The bacterial pathogen *Ralstonia solanacearum* was first cultured for 24 h, then about 200 μL (1 × 10^8^ cfu/mL) of suspension was spread on the triphenyl tetrazolium salt (TTC) medium [25]. The antagonistic strain was cultured on nutrient agar media for 24 h, then 6 mm agar blocks were cut out and placed 2.5 cm from the center. After co-culturing for 2 days at 30 °C, the inhibition zone was measured. All the experiments were performed in triplicate. The antagonistic strains were identified via 16S rRNA sequencing. A phylogenetic tree was generated using the neighbor-joining method in MEGA 11.0.

### 2.3. Indole Acetic Acid (IAA) Assay

The Salkowski’s method was used for the assay of IAA production [26]. Briefly, after the strain was cultured in King medium containing 1% L-tryptophan for 48 h, 4 mL of bacterial solution was centrifuged at 4 °C and 12,000 r/min for 10 min. Then, 2 mL of supernatant was removed, 2 mL of Salkowski coloration solution was added to the solution and mixed well, and the strain was kept in the dark at room temperature for 35 min. A UV spectrophotometer was used to measure the absorbance value of the sample at a wavelength of 530 nm.

### 2.4. Tomato Seed Germination Experiments

Based on the results of the antagonism test and IAA assay, strain YXDHD1-7 was selected to prepare a bacterial suspension, which was re-suspended using sterile water to concentrations of 10^7^ cfu/mL and 10^6^ cfu/mL. Sterile water was used as the control. Tomato seeds were soaked in 1% NaClO solution for 1 min and washed five times with sterile water. The sterilized tomato seeds were put on sterile wet filter paper in Petri dishes (10 seeds per dish) and cultured in a light incubator at 30 °C for 3 days. The control group was added with 5 mL of sterile water, and the other two treatment groups were added with 5 mL of bacterial suspension at 10^7^ cfu/mL and 10^6^ cfu/mL, respectively. After 7 days of culture, the germination rate of the tomato seeds in each treatment was calculated.

### 2.5. Antagonistic Effects on Tomato Leaves In Vitro

To minimize the sampling error, the leaflets of the third leaf were harvested from twenty-five-day-old tomato seedlings. Each group comprised twelve leaves and was first soaked for 30 min in YXDHD1-7 bacterial suspensions at 10^8^ cfu/mL, 10^7^ cfu/mL, and 10^6^ cfu/mL, respectively. Then put each four leaves into a sterile Petri dish. After that, each sterile Petri dish was sprayed five times (5 mL in total) with *A. solani* at 10^8^ cfu/mL. Sterile water (CK) and *A. solani* treatment (AS) were used as the controls [27]. All the treatments were incubated with the lid closed for 7 days (25 °C, 60% relative humidity). Changes were monitored, and the disease incidence was rated using a scale ranging from 0 to 4 [28]. The following formula was used to calculate the percent disease index (PDI): 0 = free from infection; 1 = a few spots on the leaves, covering less than 25% of the leaves’ surface area; 2 = many spots covering 25–50% of the leaves’ surface area; 3 = spots covering 51–75% of the leaves’ surface area; 4 = spots covering more than 76% of the surface area.

$$PDI\left(\%\right)=\frac{\Sigma \left(the\;number\;of\;leaves\;at\;each\;rating\;grade\times corresponding\;rating\;grade\right)\times 100}{Total\;number\;of\;leaves\times maximum\;rating\;grade}$$

$$Infection\;rate\;\left(\%\right)=\frac{Number\;of\;infected\;leaves\times 100}{Total\;number\;of\;leaves}$$

$$Relative\;control\;rate\left(\%\right)=\frac{PDI\left(AS\right)-PDI\left(treatment\right)\times 100}{PDI\left(AS\right)}$$

### 2.6. Greenhouse Bioassay

#### 2.6.1. Greenhouse Bioassay to Test Growth-Promoting Effects

Tomato seeds were disinfected with 1% NaClO for 15 min, washed several times in sterile water, and planted in a sterile seedling tray. Once 3–4 true leaves emerged, seedlings were transplanted into wet vermiculite pots along with Hoagland nutrient solution after disinfection. After 3 days, the resuspended bacterial suspension was irrigated into the tomato pot to obtain final inoculation concentrations of approximately 10^6^ cfu/g, 10^7^ cfu/g, and 10^8^ cfu/g of vermiculite, respectively. The same amount of sterile water was added to the CK group. The pot experiment was performed in a greenhouse at 28 °C and a relative humidity of 60%. After 30 days, the plant height, fresh weight per plant, and stem diameter of the tomato plants were measured. Root Analysis WINRHIZO System (Regent, Québec, QC, Canada) was used to measure the total root length and root surface area. The leaf chlorophyll concentrations were determined using a SPAD-502 meter (Konica Minolta, Tokyo, Japan).

#### 2.6.2. Greenhouse Bioassay for Biocontrol

The YXDHD1-7 bacterial suspensions (10^6^ cfu/mL, 10^7^ cfu/mL, and 10^8^ cfu/mL) were evenly sprayed onto the leaves of the treatment groups until the liquid was dripping down to the tip of the leaves. After 24 h, the same method was used to spray the tomato leaves with *A. solani* spore solution (10^8^ cfu/mL). The following groups were set up: AS (inoculated only with *A. solani* suspension at 10^8^ cfu/mL), AS + 10^6^ (inoculated with YXDHD1-7 bacterial suspension at 10^6^ cfu/mL and *A. solani* suspension at 10^8^ cfu/mL), AS + 10^7^ (inoculated with YXDHD1-7 bacterial suspension at 10^7^ cfu/mL and *A. solani* suspension at 10^8^ cfu/mL), and AS + 10^8^ (inoculated with YXDHD1-7 bacterial suspension at 10^8^ cfu/mL of and *A. solani* suspension at 10^8^ cfu/mL). The pot experiment was performed in a greenhouse at 28 °C and a relative humidity of 60%. The disease incidence was recorded at thirty days post inoculations and was rated using the previously mentioned scale, with some modifications [29]. Briefly, the plants were evaluated based on their individual disease rating grade in each treatment, where 0 = free from infection; 1 = a few spots on the leaves, covering less than 25% of the total leaves’ surface area; 2 = many spots covering 25–50% of the total leaves’ surface area; 3 = spots covering 51–75% of the total leaves’ surface area; 4 = spots covering more than 76% of the surface area. The formula used to calculate the percent disease index (PDI) is given below.

$$PDI\left(\%\right)=\frac{\Sigma \left(the\;number\;of\;plants\;at\;each\;rating\;grade\times corresponding\;rating\;grade\right)\times 100}{Total\;nunber\;of\;plants\times maximum\;rating\;grade}$$

$$Incidence\;of\;early\;blight\left(\%\right)=\frac{Number\;of\;infected\;plants\times 100}{Total\;number\;of\;plants}$$

$$Relative\;control\;rate\left(\%\right)=\frac{PDI\left(AS\right)-PDI\left(treatment\right)\times 100}{PDI\left(AS\right)}$$

### 2.7. Enzymatic Activity

Thirty days post inoculation with YXDHD1-7, the fourth leaf from the top of each plant was collected and used to determine the activity of PPO, PAL, and POD. Polyphenol oxidase (PPO) (Cat. No. PPO-1-Y), phenylalanine ammonialyase (PAL) (Cat. No. PAL-1-Y), and peroxidase (POD) (Cat. No. POD-1-Y) were determined using kits (Comin Biotechnology Co., Ltd., Suzhou, China), according to the manufacturer’s instructions. PPO activity was determined based on the method described in reference [30] using catechol as the substrate with some modifications. A variation in the absorbance at 525 nm by 0.005 per minute was defined as a unit of enzyme activity (U). PPO activity was defined as U/g fresh weight. The method reported in reference [31] was used to determine the activities of phenylalanine ammonlyase (PAL) and peroxidase (POD). A variation in the absorbance at 290 nm (or 470 nm) by 0.1 (or 0.01) per minute was defined as a unit of enzyme activity (U), and PAL (or POD) activity was expressed as U/g fresh weight.

### 2.8. Genome Sequencing and Analysis

Genomic DNA was extracted using the Bacterial DNA extraction kit (magnetic beads) (Majorbio, Shanghai, China). Sequencing libraries were generated using the NEXTFLEX Rapid DNA-Seq Kit following the manufacturer’s instructions. The draft genome of strain YXDHD1-7 was sequenced using an Illumina NovaSeq 6000 at the Majorbio Bio-Pharm Technology Co., Ltd. (Shanghai, China). A NJ (neighbor-joining) phylogenomic tree was constructed using MEGA 6.0 software using an alignment of 31 universal marker genes from 19 strains with 1000 rapid bootstrap searches [32,33]. The predicted coding DNA sequences (CDSs) were annotated from the NCBI non-redundant protein sequences (NR), Swiss-Prot, Pfam, GO, COG, and KEGG databases described in reference [34]. Gene clusters associated with the synthesis of secondary metabolites were identified using antiSMASH (Version 5.1.2). The GenBank accession number of strain YXDHD1-7 is JBBKYU000000000.

## 3. Results

### 3.1. Screening of the Antagonistic Activity of Strains and Determination of IAA Content

**Figure 1:**
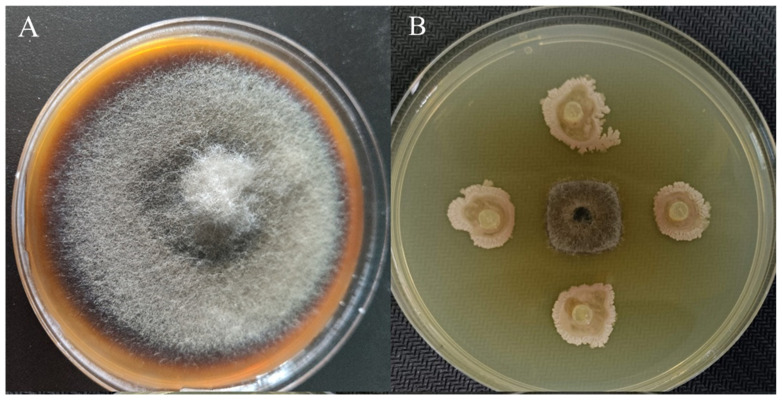
Inhibitory effect of YXDHD1-7 on *Alternaria solani* through dual-culture. (**A**), *A. solani* mycelial growth without YXDHD1-7; (**B**), *A. solani* mecelial growth with YXDHD1-7. The strains were cultured at culturing at 28 °C for 7 d.

A total of 84 pure strains were obtained from the tomato rhizosphere soil. In total, 54 of them exhibited antagonistic activity against *A. solani*. Strain YXDHD1-7 showed the strongest inhibitory effect, with an inhibition rate of 80% (Table S1, Figure 1). In addition, this strain exhibited a wide antimicrobial spectrum and was shown to exert good antagonistic effects on the pathogens of tomato bacterial wilt, pepper bacterial wilt, banana wilt, and bitter melon wilt (Table 1). This strain also produced IAA at a concentration of 25 mg/L. Based on its antagonistic ability, it was selected for subsequent growth promotion and disease control tests. The 16S rRNA gene sequence of strain YXDHD1-7 was checked against the EzBioCloud database and was revealed to be homologous to *Bacillus* strains. The phylogenic relationships between YXDHD1-7 and the other bacterial typical strains in terms of the evolutionary distances of 16S rRNA gene sequences were established based on the neighbor-joining method, as shown in Figure S1.

### 3.2. Effects of YXDHD1-7 on Tomato Seed Germination

As shown in Table 2, soaking the tomato seeds in the YXDHD1-7 culture had a positive effect on their germination at 10^6^ cfu/mL. The germination rate did not differ significantly between the control and treated groups, but the YXDHD1-7 treatment promoted the bud and root lengths in the seedlings. Under inoculation at 10^6^ cfu/mL, these two parameters increased significantly by 62.94% and 112.80%, respectively, compared with the values observed in the control. Inoculation at 10^7^ cfu/mL increased root length by 36.10%. The results showed that the selected strain had no significant effect on the seed germination rate but promoted bud and root lengths in seedlings.

### 3.3. Effects of the In Vitro YXDHD1-7 Treatment on Detached Infected Tomato Leaves

**Figure 2:**
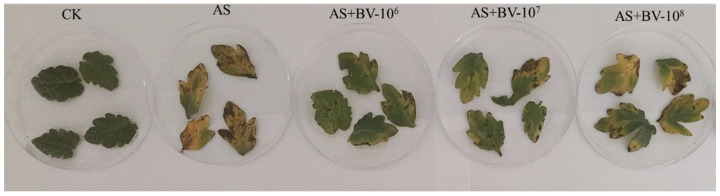
Biocontrol effect of strain YXDHD1-7 on detached infected tomato leaves. CK: sterile water treatment; AS: treatment with *A. solani*; AS + BV-10^6^, AS + BV-10^7^, and AS + BV-10^8^: treatments with both *A. solani* and *B. velezensis* (YXDHD1-7) suspensions at 10^6^ cfu/mL, 10^7^ cfu/mL, and 10^8^ cfu/mL, respectively.

Detached tomato leaves with different YXDHD1-7 inoculation concentrations with *A. solani* were placed in an incubator at 25 °C and 60% relative humidity for 7 days to observe the incidence of early blight disease. The selected strain significantly inhibited pathogenesis in the leaves (Figure 2). The percent disease index in all the treatment groups was lower than that in the control group (Table 3). Among the YXDHD1-7 treatments, that at 10^6^ cfu/mL was the best, with an inhibition rate of 81.39%, followed by those at 10^7^ cfu/mL (56.93%) and 10^8^ cfu/mL (12.72%). These results suggested that the selected strain exerts an antagonistic effect on *A. solani* that is visible in detached infected leaves.

### 3.4. Greenhouse Bioassay for Growth Promotion and the Ability of YXDHD1-7 to Prevent Disease

#### 3.4.1. Effect of Strain YXDHD1-7 on Tomato Seedling Growth

**Figure 3:**
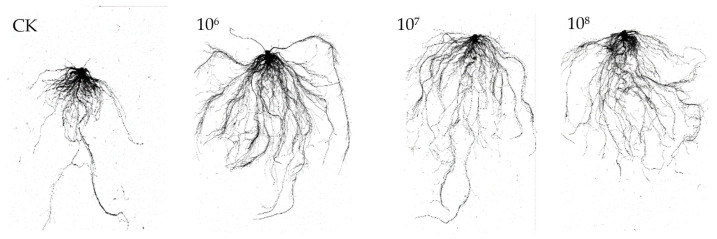
Effects of inoculation with strain YXDHD1-7 on tomato root growth. 10^6^: YXDHD1-7 suspension at 10^6^ cfu/g vermiculite; 10^7^: YXDHD1-7 suspension at 10^7^ cfu/g vermiculite; 10^8^: YXDHD1-7 suspension at 10^8^ cfu/g vermiculite.

The pot experiment to test growth promotion using the selected strain showed that 30 days after inoculation, plant height, stem diameter, fresh weight per plant, total root length, and root surface area in the treated groups were significantly higher than those in the CK group. Figure 3 shows the growth-promoting effect of the selected strain on tomato roots at different inoculation concentrations. Among them, the treatment at 10^6^ cfu/g vermiculite had the most significant effect. Specifically, compared with the control, the fresh weight per plant, total root length, stem diameter, plant height, root surface area, and chlorophyll content increased by 81.83%, 194.45%, 27.91%, 26.21%, 152.65%, and 39.52% (Table 4), respectively.

#### 3.4.2. Effects of Strain YXDHD1-7 on Tomato Early Blight

Inoculation with the selected strain significantly inhibited the occurrence of tomato early blight (Table 5). Thirty days post inoculation, the disease incidence was effectively reduced by the treatments at different YXDHD1-7 concentrations. Specifically, inoculations at 10^6^, 10^7^, and 10^8^ cfu/mL of the selected strain resulted in control efficacies of 100%, 83.15%, and 69.9%, respectively. The YXDHD1-7 suspensions at all the tested concentrations promoted chlorophyll content in the tomato plants. Among them, the bacterial solution at 10^6^ cfu/mL had the best control effect. The above results showed that inoculation with the selected strain significantly reduced the incidence of tomato early blight.

#### 3.4.3. Effects of Strain YXDHD1-7 on the Activity of Defense Enzymes in Tomato Leaves

**Figure 4:**
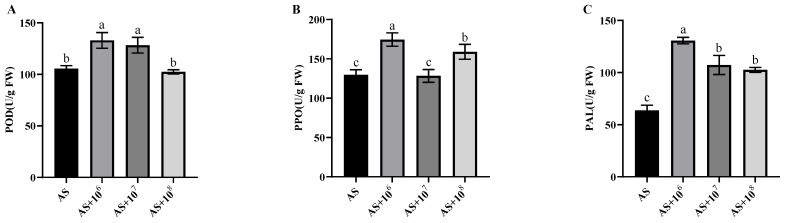
Effect of strain YXDHD1-7 on the response of tomato plants to *A. solani* infection. (**A**–**C**) Activities of POD, PPO, and PAL in tomato leaves, respectively. The values were measured 30 days after transplant. Peroxidase (POD), polyphenol oxidase (PPO), and phenylalanine deaminase (PAL). Significant differences are marked with letters (*p* < 0.05).

As shown in Figure 4, the activities of POD, PPO, andPAL significantly increased by 25.93%, 34.36%, and 104.99%, respectively, in plants inoculated with the suspension at 10^6^ cfu/mL. When the concentration increased to 10^7^ cfu/mL, the activities of POD and PAL significantly increased by 21.53%% and 68.31%, respectively, while that of PPO did not vary significantly. At the highest concentration of 10^8^ cfu/mL, the activities of PPO and PAL also significantly increased by 22.41% and 60.92%%, respectively. The above results showed that inoculation with the YXDHD1-7 suspension effectively enhanced the activities of these enzymes in tomato plants, especially at 10^6^ cfu/mL, thereby improving their resistance to early blight.

### 3.5. Genome Sequencing of Strain YXDHD1-7

#### 3.5.1. Genomic Features

**Figure 5:**
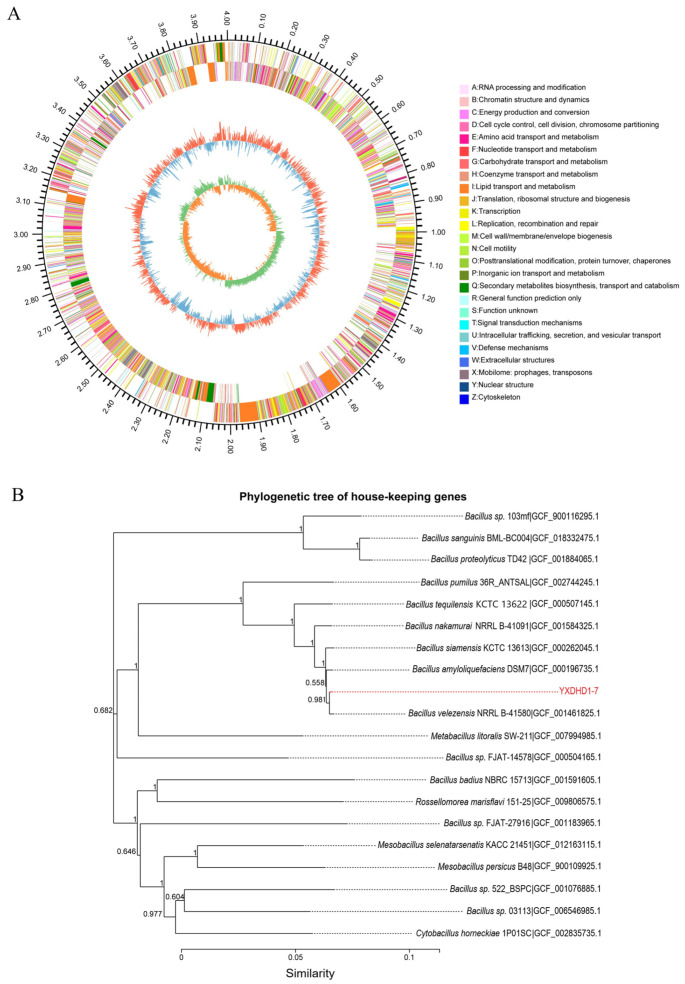
Genome map of strain YXDHD1-7 (**A**) and its phylogenetic taxonomic position (**B**). (**A**) Genome map showing the features of strain YXDHD1-7. The circles from the outside to the inside denote protein-coding sequences, rRNA, tRNA, GC content, positive and negative GC skew. (**B**) NJ phylogenomic tree with bootstrap support inferred based on 31 house-keeping genes. Bootstrap values expressed as percentages of 1000 replications.

After assembly, the draft genome size of strain YXDHD1-7 was 4007,168 bp, with a GC content of 46.4%. It possessed a total of 3891 genes, with 83 tRNAs and 7 rRNAs. The predicted genes included 1882 genes involved in metabolism, 289 genes involved in environmental information processing, and 166 genes involved in cellular processes. Functional classification based on the COG database was used to identify genes involved in carbohydrate transport and metabolism (*n* = 273), transcription (*n* = 300), general functions (*n* = 251), and signal transduction (*n* = 200). A total of 128 carbohydrate-active enzyme-encoding genes were identified in YXDHD1-7, including glycosyl hydrolysis-related enzymes (GHs, 32.0%), glycosyl transferases (GTs, 32.0%), carbohydrate esterases (CEs, 25.0%), carbohydrate-binding modules (CBMs, 1.6%), polysaccharide lyases (PLs, 2.3%), and enzymes related to auxiliary activities (AAs, 7.0%). The circular genome of the selected strain was visualized using the circular viewer and is shown in Figure 5A. To evaluate the phylogenetic position of YXDHD1-7, we created an NJ phylogenetic tree based on the alignment of nucleotide sequences for the 31 house-keeping genes, which showed that the strain and *B. velezensis* were clustered together (Figure 5B). Thus, YXDHD1-7 was identified as *B. velezensis*.

#### 3.5.2. Genetic Basis for the Plant Growth-Promoting and Anti-Pathogen Effects of YXDHD1-7

The genes encoding IAA, spermidine, and polyamine as well as the volatile compound were identified in *B. velezensis* YXDHD1-7, which have been reported to play key roles in promoting plant growth (Table 6). It was reported that volatile compounds produced by PGPRs can induce ISR against various plant pathogens [19,35,36], and the spermidine was reported to have a protective effect on chlorophyll content under stress [37]. In addition, this strain was shown to possess 14 genes involved in biofilm formation, development, and regulation. The representative gene clusters encoding putative secondary metabolites are summarized in Table 7. The analysis of its genome identified 22 gene clusters encoding secondary metabolites. The putative natural products included bacteriocin, lanthipeptide, terpene, polyketide sysnthases-like (PKS-like), nonribosomal peptide synthetases (NRPS), transAT-PKS, type I PKS, and type III PKS. The NRPS contained some peptide antibiotics, such as fengycin, plipastatin, and surfactin.

## 4. Discussion

Tomato plants are particularly susceptible to early blight caused by *A. solani*, which is responsible for significant losses in production [38]. In recent years, *B. velezensis* has been studied due to its plant growth-promoting and biocontrol properties, and it is considered to have great potential for application in agriculture [39,40,41]. In this study, an IAA-producing strain YXDHD1-7 (*B. velezensis*), which had the strongest inhibitory effect against the pathogen, was effective in reducing the incidence of tomato early blight. This bacterium may secrete secondary metabolites that directly inhibit the early blight pathogen through the synthesis of antimicrobial metabolites. It was reported that the most common volatile compound types released by *B. velezensis*, such as ketones, alcohols, alkanes, pyrazine, and benzothiazole, exhibited significant antifungal activities against *A. solani* [42,43]. In addition, non-volatile lipopeptides and volatiles ketone metabolites secreted by *B. velezensis* were also shown to have inhibitory effects on *A. solani* [44]. *B. velezensis* strains are considered to be a treasure house of bioactive compounds for biocontrol, and these metabolites are strain-specific. Therefore, genome surveys are necessary to describe the possible mechanisms through which these different strains promote plant growth and exert biocontrol effects. Gene clusters of fengycin, surfactin, and bacilysin that are responsible for antifungal metabolites have been identified in *B. velezensis* [45]. The predicted bioactive metabolites in strain YXDHD1-7 include bacteriocin, lanthipeptide, terpene, NRPS, PKS-like, transAT-PKS, T1PKS, T3PKS, and transAT-PKS-like, among others (Table 7). It comprises four gene clusters associated with fengycin and surfactin, which may play key roles in the inhibiting growth of *A. solani*. Additionally, this strain likely secrets other new bioactive compounds, as it comprises the lanthipeptide biosynthetic gene cluster.

*B. velezensis* could prevent disease by inducing resistance and promoting the growth of plants [44,46]. For example, it has been reported that the application of *B. velezensis* controlled crown gall disease in *Prunus subhirtella* by increasing the activities of PPO and PAL [47]. The lipopeptides released by *B. velezensis* were reported to elicit defense responses against *Fusarium verticillioides* in maize seedlings [48], while *B. velezensis* YYC was shown to enhance the activity of defense-related enzymes in response to bacterial wilt infection [49]. In this study, the bacterial suspension of YXDHD1-7 not only significantly promoted root and bud lengths in seedlings but also significantly enhanced chlorophyll content, plant height, fresh weight, and stem diameter in tomato plants (Table 4). Additionally, this strain was shown to promote the activities of defense enzymes (POD, PAL, and PPO) in tomato leaves (Figure 4). The volatile compounds elicited by *Bacillus* spp., such as 2,3-butanediol and acetoin (3-hydroxy-2-butanone), were able to promote plant growth and induce systemic resistance (ISR) [50,51,52]. The acetoin of *B. velezensis* was found to increase the host plant defense response enzymes activity (e.g., POD, PAL, and PPO) [20]. A genomic analysis revealed that the selected strain possessed the genes involved in the production of acetoin, which may contribute to the properties of PGPR and the defense response stimulator (Table 6) [53,54]. Our laboratory experiments also demonstrated that *B. velezensis* YXDHD1-7 could produce IAA and possesses genes that are involved in the production of IAA and spermidine. Exogenous spermidine increased the chlorophyll content by down–regulating the chlorophyllase gene expression in a saline-stressed environment [53]. It was reported that the application of *B. velezensis* could enhance the growth of nine selected plants by producing IAA to promote growth in peanut plants [45,55]. However, our study is the first systematic report demonstrating that *B. velezensis* prevented tomato early blight by promoting growth and enhancing defense enzyme activities. It was observed that the plant growth-promoting or biocontrol effects of the YXDHD1-7 inoculation at 10^6^ cfu/g vermiculite or cfu/mL were considerably higher than those at the other two tested concentrations (10^7^ and 10^8^ cfu/g vermiculite or cfu/mL), indicating that the inoculation needs to be at the right concentration in order to be more effective. This strain is a promising candidate for further testing and development followed by field trials to obtain excellent microbial inoculants that can be used to enhance tomato production.

## 5. Conclusions

In this study, strain YXDHD1-7 was isolated from tomato rhizosphere soil and identified as *B. velezensis*. The strain exhibited a broad-spectrum anti-fungal or anti-bacterial activity and could effectively increase root and bud lengths in tomato seedlings. Inoculation with YXDHD1-7 effectively increased the chlorophyll content and physiological indexes of the tomato plants and had a significant growth-promoting effect. It was proved that the strain enhanced the resistance of the tomato plants to early blight by regulating the activity of defense enzymes. In terms of its genetic features, strain YXDHD1-7 was shown to possess 22 gene clusters associated with the synthesis of anti-fungal or anti-bacterial metabolites. Its PGPR activity was attributed to genes involved in the production of IAA, spermidine, polyamine, volatile compounds, and biofilm formation. It was observed that the plant growth-promoting or biocontrol effects of the YXDHD1-7 inoculation at different concentrations varied significantly, indicating that the inoculation needs to be performed at the right concentration in order to be more effective. However, as the dose trials have been repeated only once and bacterial concentrations below 10^6^ cfu/mL of strain were not tested, further optimization experiments are needed to explore the best inoculation concentration in the field trials.

## Figures and Tables

**Table 1:** Indole acetic acid yield and antagonistic effect of YXDHD1-7 on pathogenic bacteria or fungi.

IAA (mg/L)	*A. asolani*	*F. oxysporum* f. sp. *momordicae*	*F. oxysporum* f. sp. *cubense*	*R. solanacearum* (Tomato)	*R. solanacearum* (Pepper)
25.51 ± 1.55	Inhibition rate (%)			Inhibition zone (mm)	
	80.08 ± 0.15	52.01 ± 1.08	54.27 ± 2.27	19.34 ± 0.13	14.20 ± 1.29

Note: values are presented as mean ± SD of three replications.

**Table 2:** Effects of YXDHD1-7 on tomato seed germination.

Treatments	Bud Length (mm)	Root Length (mm)	Germination Rate (%)
CK	9.39 ± 2.29 b	18.90 ± 6.98 c	93.33 ± 5.77 a
10^6^	15.30 ± 4.12 a	40.22 ± 10.03 a	96.67 ± 5.77 a
10^7^	9.62 ± 3.79 b	25.72 ± 9.56 b	90.00 ± 0.01 a

Note: values are presented as mean ± SD of thirty tomato seeds. CK indicates the seed soaked in the sterilized water; 10^6^ or 10^7^ treatment indicates the seed soaked in the YXDHD1-7 suspension at 10^6^ or 10^7^ cfu/mL. The tomato seeds were cultured in a light incubator at 30 °C for 3 days. Significant differences are marked with letters (*p* < 0.05).

**Table 3:** Inhibitory effects of YXDHD1-7 on detached infected tomato leaves.

Treatment	Infection Rate (%)	Percent Disease Index (PDI) (%)	Relative Control Rate (%)
AS	100.00 ± 0	99.67 ± 0.47	-
AS + BV-10^6^	72.50 ± 2.5	18.42 ± 0.31	81.39 ± 0.14
AS + BV-10^7^	100.00 ± 0	42.25 ± 1.62	56.93 ± 0.63
AS + BV-10^8^	100.00± 0	86.50 ± 1.08	12.72 ± 0.53

Note: values are presented as mean ± SD of twelve detached infected leaves. The detached tomato leaves were first soaked for 30 min in bacterial suspensions and then sprayed five times (5 mL in total) with *A. solani*. All the treatments were incubated at room temperature with the lid closed for 7 days (25 °C, 60% relative humidity). AS indicates the treatment with *A. solani*; AS + BV-10^6^, AS + BV-10^7^, and AS + BV-10^8^ indicate the treatments with both *A. solani* and *B. velezensis* YXDHD1-7 suspensions at 10^6^ cfu/mL, 10^7^ cfu/mL, and 10^8^ cfu/mL, respectively.

**Table 4:** Growth-promoting effects of strain YXDHD1-7 on tomato plants.

Treatment	Plant Fresh Weight (g)	Total Root Length (cm)	Stem Diameter (cm)	Plant Height (cm)	Root Surface Area (cm^2^)	Chlorophyll Content (SPAD)
CK	5.12 ± 0.26 c	375.82 ± 9.27 c	0.43 ± 0.14 c	14.00 ± 0.50 c	39.56 ± 0.31 c	37.20 ± 0.49 d
10^6^	9.31 ± 1.03 a	1106.62 ± 14.61 a	0.55 ± 0.23 a	17.67 ± 0.58 a	99.95 ± 1.66 a	51.90 ± 1.41 a
10^7^	6.70 ± 0.67 b	780.09 ± 79.63 b	0.51 ± 0.36 ab	16.00 ± 0.50 b	83.92 ± 6.85 b	41.17 ± 2.24 c
10^8^	7.77 ± 0.09 b	1100.93 ± 36.67 a	0.49 ± 0.09 b	16.00 ± 1.00 b	100.17 ± 8.04 a	46.33 ± 2.06 b

Note: values are presented as mean ± SD of six replications. 10^6^, 10^7^, and 10^8^ indicate YXDHD1-7 inoculations at 10^6^, 10^7^, and 10^8^ cfu/g vermiculite. The values were measured 30 days after transplant. Significant differences are marked with letters (*p* < 0.05).

**Table 5:** Inhibitory effects of YXDHD1-7 on tomato early blight.

Treatment	Percent Disease Index (PDI) (%)	Relative Control Rate (%)	Chlorophyll Content (SPAD)
AS	100.00 ± 0	-	28.65 ± 0.85 d
AS + 10^6^	0 ± 0	100.00 ± 0	45.20 ± 0.87 a
AS + 10^7^	16.35 ± 0.35	83.15 ± 0.15	39.35 ± 0.9 c
AS + 10^8^	28.60 ± 0.6	69.90 ± 0.9	41.43 ± 0.93 b

Note: values are presented as mean ± SD of six replications. AS indicates the treatment inoculated only with *A. solani* suspension; AS + 10^6^, AS + 10^7^, or AS + 10^8^ indicates the treatment inoculated both YXDHD1-7 bacterial suspension at 10^6^,10^7^, or 10^8^ cfu/mL and *A. solani*. The values were measured 30 days after transplant. Significant differences are marked with letters (*p* < 0.05).

**Table 6:** Genes identified in the genome of *B. velezensis* YXDHD1-7 predicted to be involved in plant growth promotion.

Gene Name	Length (bp)	Swiss-Prot Description	Organisms Comprising Orthologues Genes
Putative IAA production-related genes in *B. velezensis* YXDHD1-7			
*trpA*	798	Tryptophan synthase alpha chain	*B. velezensis* (strain DSM 23117/BGSC 10A6/LMG 26770/FZB42)
*trpB*	1203	Tryptophan synthase beta chain	*B. velezensis* (strain DSM 23,117/BGSC 10A6/LMG 26770/FZB42)
*trpC*	753	Indole-3-glycerol phosphate synthase	*B. velezensis* (strain DSM 23117/BGSC 10A6/LMG 26770/FZB42)
*trpD*	1017	Anthranilate phosphoribosyltransferase	*B. velezensis* (strain DSM 23117/BGSC 10A6/LMG 26770/FZB42)
*trpE*	1548	Anthranilate synthase component 1	OS = *B. subtilis* (strain 168)
*trpF*	654	N-(5’-phosphoribosyl) anthranilate isomerase	*B. velezensis* (strain DSM 23117/BGSC 10A6/LMG 26770/FZB42)
Putative spermidine and polyamine production-related genes in *B. velezensis* YXDHD1-7			
*msmX1*	1107	Uncharacterized ABC transporter ATP-binding protein YurJ	*B. subtilis* (strain 168)
*msmX2*	1101	Oligosaccharides import ATP-binding protein MsmX	*B. subtilis* (strain 168)
*pksS*	1212	Polyketide biosynthesis cytochrome P450 PksS	*B. subtilis* (strain 168)
*speE*	831	Polyamine aminopropyltransferase	*B. subtilis* (strain 168)
*speG*	459	Spermine/spermidine N (1)-acetyltransferase	*B. subtilis* (strain 168)
Putative volatile compound production-related genes in *B. velezensis* YXDHD1-7			
*alsD*	768	Alpha-acetolactate decarboxylase	*B. subtilis* (strain 168)
*ilvB*	1716	Acetolactate synthase	*B. subtilis* (strain 168)
*bdhA*	807	Uncharacterized oxidoreductase YxjF	*B. subtilis* (strain 168)
*bdhA*	786	Uncharacterized oxidoreductase YxjF	*B. subtilis* (strain 168)
*ilvB*	1725	Acetolactate synthase large subunit	*B. subtilis* (strain 168)
*acuC*	1167	Acetoin utilization protein AcuC	*B. subtilis* (strain 168)
*gapA*	1008	Glyceraldehyde-3-phosphate dehydrogenase 1	*Bacillus subtilis* (strain 168)
*gapA*	1023	Glyceraldehyde-3-phosphate dehydrogenase 2	*Bacillus subtilis* (strain 168)
Putative biofilm formation, development, and regulation-related genes in *B. velezensis* YXDHD1-7			
*iolU*	987	Scyllo-inositol 2-dehydrogenase (NADP(+)) IolU	*B. subtilis* (strain 168)
*rpoN*	1311	RNA polymerase sigma-54 factor	*B. subtilis* (strain 168)
*slrR*	456	HTH-type transcriptional regulator SlrR	*B. subtilis* (strain 168)
*csrA*	225	Translational regulator CsrA	*B. velezensis* (strain DSM 23117/BGSC 10A6/LMG 26770/FZB42)
*flgM*	267	Negative regulator of flagellin synthesis	*B. subtilis* (strain 168)
*wecB*	1140	UDP-N-acetylglucosamine 2-epimerase	*B. subtilis* (strain 168)
*tagA*	771	N-acetylglucosaminyldiphosphoundecaprenol N-acetyl-beta-D-mannosaminyltransferase	*B. subtilis* (strain 168)
*cysE*	654	Serine acetyltransferase	*B. subtilis* (strain 168)
*fliA*	765	RNA polymerase sigma-D factor	*B. subtilis* (strain 168)
*hfq*	222	RNA-binding protein Hfq	*B. velezensis* (strain DSM 23117/BGSC 10A6/LMG 26770/FZB42)
*sinR*	342	HTH-type transcriptional regulator SinR	*B. subtilis* (strain 168)
*luxS*	474	S-ribosylhomocysteine lyase	*B. velezensis* (strain DSM 23117/BGSC 10A6/LMG 26770/FZB42)
*crr*	507	Putative phosphotransferase enzyme IIA component YpqE	*B. subtilis* (strain 168)
*trpE*	1548	Anthranilate synthase component 1	*B. subtilis* (strain 168)

**Table 7:** Putative gene clusters encoding secondary metabolites in *B. velezensis* YXDHD1-7.

Cluster ID	Type	Similar Cluster	Similarity (%)	MIBiG Accession
1	Bacteriocin	Amylocyclicin	100	BGC0000616
2	Other	Bacilysin	100	BGC0001184
3	Lanthipeptide	Mersacidin	100	BGC0000527
4	PKS-like	Butirosin A/butirosin B	7	BGC0000693
5	terpene	-	-	-
6	TransAT-PKS	Macrolactin H	100	BGC0000181
7	TransAT-PKS	Bacillaene	100	BGC0001089
8	NRPS	Fengycin	86	BGC0001095
9	TransAT-PKS	Difficidin	46	BGC0000176
10	NRPS	Plipastatin	53	BGC0000407
11	Terpene	-	-	-
12	T3PKS	-	-	-
13	TransAT-PKS-like	Difficidin	53	BGC0000176
14	NRPS	Surfactin	39	BGC0000433
15	NRPS	Surfactin	47	BGC0000433
16	T1PKS	Macrobrevin	26	BGC0001470
17	T3PKS	Myxovirescin A1	17	BGC0001025
18	TransAT-PKS-like	Difficidin	26	BGC0000176
19	NRPS	-	-	-
20	NRPS	Fengycin	13	BGC0001095
21	TransAT-PKS-like	Bryostatin	80	BGC0000174
22	TransAT-PKS-like	-	-	-

## Data Availability

The raw data supporting the conclusions of this article will be made available by the authors on request.

## Supplementary Materials

The following supporting information can be downloaded at: https://www.mdpi.com/article/10.3390/microorganisms12050921/s1, Figure S1: Phylogenetic tree illustrating the relationships between YXDHD1-7 and the examined bacterial strains in EzBioCloud in terms of the evolutionary distances of 16S rRNA gene sequences using the neighbor-joining method; Table S1: Antagonistic effect of isolated strains on *A. asolani*.
