# Supplementary material for: Bacillus velezensis YXDHD1-7 Prevents Early Blight Disease by Promoting Growth and Enhancing Defense Enzyme Activities in Tomato Plants
Source: Microorganisms. 2024 Apr 30;12(5):921. doi: 10.3390/microorganisms12050921 (PMC11124510; doi:10.3390/microorganisms12050921)
Supplement: Supplementary file 1 [file microorganisms-12-00921-s001.zip › microorganisms-2975334-supplementary.pdf]

Table S1. Antagonistic effect of isolated strains on *A. asolani*

Strain No.	Sampling location	Inhibition rate (%)	16S r DNA Identification	Similarity (%)
YXDHD1-7	Yangjiang	80.00±0.16	*Bacillus velezensis*	99.86
YXPT1-4-1	Yangjiang	76.22±0.99	*Bacillus siamensis*	99.86
YXDHD1-4-2	Yangjiang	75.79±0.83	*Bacillus velezensis*	99.99
YXDHD1-6-2	Yangjiang	74.54±0.01	*Bacillus siamensis*	99.60
DBBHT1-8-1	Maoming	74.13±0.69	*Bacillus siamensis*	99.60
YXDHD1-3-1	Yangjiang	73.37±0.17	*Bacillus siamensis*	99.90
DBBHT2-2	Maoming	72.55±0.09	*Bacillus siamensis*	99.60
DBBHT2-3-1	Maoming	72.54±0.08	*Bacillus siamensis*	99.86
YXDHD1-6-1	Yangjiang	70.85±0.94	*Bacillus siamensis*	99.60
YXDHD1-5	Yangjiang	70.41±0.76	*Bacillus siamensis*	99.64
YXDHD1-7-1	Yangjiang	70.06±0.33	*Bacillus siamensis*	99.64
YXXT2-2-2	Yangjiang	69.55±0.97	*Bacillus siamensis*	99.99
DBSDH3	Maoming	69.03±0.42	*Bacillus siamensis*	99.90
DBDY-1-5-3	Maoming	68.75±0.32	*Bacillus siamensis*	99.60
DBSDH5	Maoming	68.02±0.47	*Bacillus siamensis*	99.60
DBSD2-7-1	Maoming	65.61±0.51	*Bacillus tequilensis*	99.93
DBBHT1-3-1	Maoming	64.81±0.74	*Bacillus velezensis*	99.93
DBSDH6-1	Maoming	63.07±0.99	*Bacillus velezensis*	99.93
DBBHT1-6	Maoming	62.77±7.41	*Paenibacillus glycanilyticus*	99.60
DBGE1-11	Maoming	62.6±2.38	*Streptomyces acidiscabies*	99.60
YXDHD1-4-6	Yangjiang	62.57±0.47	*Bacillus siamensis*	99.93
DBSD2-6	Maoming	62.38±0.16	*Bacillus tequilensis*	99.90
DBGE1-3	Maoming	61.18±1.44	*Bacillus velezensis*	99.85
YXPT1-1-1	Yangjiang	60.17±0.99	*Bacillus tequilensis*	99.93
DBSD2-6-1	Maoming	59.96±0.28	*Bacillus tequilensis*	99.93
DBGE1-3-1	Maoming	59.47±0.33	*Bacillus velezensis*	99.93
DBHHT1-6-1	Maoming	56.32±5.51	*Paenibacillus glycanilyticus*	99.30
DBBHT1-2	Maoming	55.97±1.38	*Bacillus siamensis*	100
DBBHT1-5	Maoming	55.04±0.24	*Bacillus siamensis*	99.51

DBKZ2-1-1	Maoming	53.51±0.43	*Paenibacillus cineris*	99.38
YXXT1-3	Yangjiang	53.27±0.72	*Bacillus altitudinis*	99.79
YXDHD1-7-2	Yangjiang	52.58±1.68	*Bacillus altitudinis*	99.79
DBSDH6	Maoming	52.1±0.63	*Bacillus velezensis*	100
YXXT1-8	Yangjiang	51.19±0.04	*Paenibacillus cineris*	99.24
DBKZ1-3-1	Maoming	51.13±1.7	*Mesobacillus thioparans*	99.79
DBBHT2-3	Maoming	51.06±1.53	*Metabacillus idriensis*	100
DBDY-1-7-5	Maoming	50.21±0.01	*Bacillus altitudinis*	99.79
YXDHD1-1	Yangjiang	48.94±1.28	*Streptomyces griseorubiginosus*	99.90
YXDHD1-3-2	Yangjiang	48.83±0.41	*Bacillus rhizoplanae*	99.56
DBKZ1-5	Maoming	48.53±0.39	*Bacillus altitudinis*	100
DBKZ1-3-2	Maoming	48.29±0.26	*Bacillus rhizoplanae*	99.50
DBBHT1-8-3	Maoming	47.39±1.71	*Bacillus altitudinis*	99.79
YXXT1-8-2	Maoming	47.39±1.21	*Bacillus siamensis*	99.86
DBDY1-4	Maoming	45.94±0.42	*Kitasatospora aureofaciens*	98.85
DBSD2-1-4	Maoming	45.51±1.31	*Bacillus subtilis*	99.71
DBSDH3-1	Maoming	45.25±0.25	*Mesobacillus thioparans*	99.43
DBKZ1-4-1	Maoming	45.1±0.88	*Staphylococcus haemolyticus*	99.79
DBKZ2-2	Maoming	43.61±0.54	*Streptomyces fodineus*	99.36
DBDY-1-4	Maoming	43.44±0.27	*Priestia megaterium*	99.44
DBKZ1-3	Maoming	42.42±0.15	*Staphylococcus haemolyticus*	100
DBGE1-2-1	Maoming	38.42±0.5	*Bacillus altitudinis*	100
DBSD1-11	Maoming	55.17±0.67	*Streptomyces albogriseolus*	100
DBBHT1-7	Maoming	47.31±0.95	*Streptomyces seoulensis*	99.49
DBDY1-3	Maoming	36.26±0.36	*Streptomyces griseoruber*	100

Notice: Inhibition rate values are presented as mean ± SD of three replications. In fact, *Bacillus* strains can only be identified to the genus level using 16S rRNA.

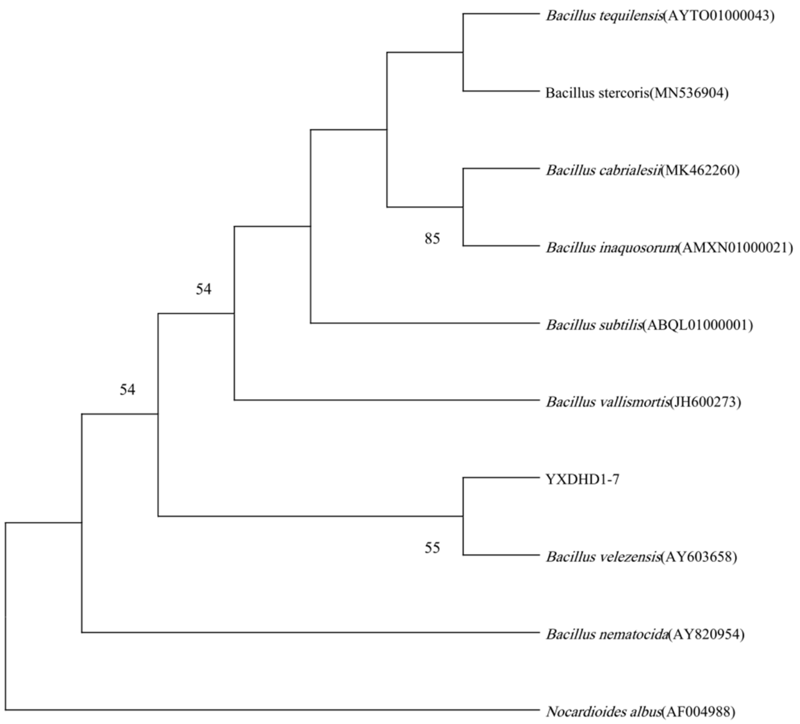

Figure S1. Phylogenetic tree illustrating the relationships between YXDHD1-7 and the examined bacterial strains in EzBioCloud in terms of the evolutionary distances of 16S rRNA gene sequences using the neighbor-joining method.
